# Weakly supervised video-based cardiac detection for hypertensive cardiomyopathy

**DOI:** 10.1186/s12880-023-01035-0

**Published:** 2023-10-19

**Authors:** Jiyun Chen, Xijun Zhang, Jianjun Yuan, Renjie Shao, Conggui Gan, Qiang Ji, Wei Luo, Zhi-Feng Pang, Haohui Zhu

**Affiliations:** 1 Department of Ultrasonography, Henan Provincial People’s Hospital, Zhengzhou, 450003 China; 2 Research & Development Center, CHISON Medical Technologies Co., Ltd, Wuxi, 214142 China; 3 University of Chinese Academy of Sciences, Beijing, 100049 China; 4 School of Mathematics and Statistics, Henan University, Kaifeng, 475000 China

**Keywords:** Three-dimensional (3-D) convolutional neural network, Artificial intelligence, Assisted diagnosis, Echocardiographic video, Hypertensive cardiomyopathy

## Abstract

**Introduction:**

Parameters, such as left ventricular ejection fraction, peak strain dispersion, global longitudinal strain, etc. are influential and clinically interpretable for detection of cardiac disease, while manual detection requires laborious steps and expertise. In this study, we evaluated a video-based deep learning method that merely depends on echocardiographic videos from four apical chamber views of hypertensive cardiomyopathy detection.

**Methods:**

One hundred eighty-five hypertensive cardiomyopathy (HTCM) patients and 112 healthy normal controls (N) were enrolled in this diagnostic study. We collected 297 de-identified subjects’ echo videos for training and testing of an end-to-end video-based pipeline of snippet proposal, snippet feature extraction by a three-dimensional (3-D) convolutional neural network (CNN), a weakly-supervised temporally correlated feature ensemble, and a final classification module. The snippet proposal step requires a preliminarily trained end-systole and end-diastole timing detection model to produce snippets that begin at end-diastole, and involve contraction and dilatation for a complete cardiac cycle. A domain adversarial neural network was introduced to systematically address the appearance variability of echo videos in terms of noise, blur, transducer depth, contrast, etc. to improve the generalization of deep learning algorithms. In contrast to previous image-based cardiac disease detection architectures, video-based approaches integrate spatial and temporal information better with a more powerful 3D convolutional operator.

**Results:**

Our proposed model achieved accuracy (ACC) of 92%, area under receiver operating characteristic (ROC) curve (AUC) of 0.90, sensitivity(SEN) of 97%, and specificity (SPE) of 84% with respect to subjects for hypertensive cardiomyopathy detection in the test data set, and outperformed the corresponding 3D CNN (vanilla I3D: ACC (0.90), AUC (0.89), SEN (0.94), and SPE (0.84)). On the whole, the video-based methods remarkably appeared superior to the image-based methods, while few evaluation metrics of image-based methods exhibited to be more compelling (sensitivity of 93% and negative predictive value of 100% for the image-based methods (ES/ED and random)).

**Conclusion:**

The results supported the possibility of using end-to-end video-based deep learning method for the automated diagnosis of hypertensive cardiomyopathy in the field of echocardiography to augment and assist clinicians.

**Trial registration:**

Current Controlled Trials ChiCTR1900025325, Aug, 24, 2019. Retrospectively registered.

## Introduction

As an important risk factor for the cardiovascular disease [1], the hypertension (HTN) is a common disease with a high prevalence in the world [2]. At the same time, the HTN is also a disease that develops silently and without acute clinical symptoms. Especially, delay in the therapy of HTN patients is common, leading to their suffering from serious cardiovascular events [3]. Thus, the early identification of preclinical cardiac damage caused by the HTN is crucial for the early intervention to improve the prognosis. Sustained HTN induces left ventricular (LV) remodeling and hypertrophy, which widely influence LV systolic and diastolic functions [4, 5]. Left ventricular ejection fraction (LVEF) by conventional echocardiography is not sensitive enough to detect early subclinical LV dysfunction, particularly in patients with preserved LVEF [6, 7]. Multiple studies have recently shown that speckle tracking echocardiography (STE) is valuable for evaluating cardiac function [8, 9]. However, this method is subjective and also depends on the medical specialist's experience [10]. In contrast, machine learning uses computers to automatically improve the outcomes. It is one of today’s most rapidly growing technical fields, lying at the intersection of computer science and statistics, and at the core of artificial intelligence and data science [11]. As one of the main techniques in the field of the machine learning, the convolutional neural networks (CNNs) have been dominant in a variety of computer vision tasks, leading to create new powerful architectures constantly and enhance the accuracies or benchmarks of public datasets. Nevertheless, few studies have applied deep learning algorithms to discriminate controls from patients affected by cardiovascular diseases via echocardiographic data explicitly [12, 13] or implicitly [14]. Especially, the majority of them are image-based and neglected possible temporal effects that may contribute to the final diagnosis to a certain extent. Explicit methods [12, 13, 15] straightforwardly train a two-dimensional (2-D) CNN to infer the existence of myocardiopathy from images. Implicit methods [14] can assess cardiac function through ejection fraction derived from a deep learning pipeline, integrating tasks of segmenting the LV through using weak supervision to determine cardiac cycles and average the ejection fraction predictions for each ventricular beat throughout the entire video estimated by a spatiotemporal three-dimensional (3-D) CNN. The current 3-D CNNs, aiming to resolve video-based classification problems, heavily rely on trimmed videos for the model training [16].

In the present study, solely untrimmed echocardiographic videos and their corresponding myocardiopathy types were obtained from clinics, and the cardiovascular disease detection was regarded as a weakly supervised problem since the problematic part of a video was unknown, providing that the video-based ground truth label might appear as a certain disease type. This is to say, using a patient's echo video, we could exactly indicate which clip or consecutive frames could contribute to the diagnosis of certain cardiac diseases, while we only were aware of the diagnostic results of the whole video. This study aimed to incorporate the attributes of echocardiographic videos, weakly supervised methods based on multiple-instance learning (MIL) [16], domain adversarial neural networks, and 3-D CNNs to detect hypertensive cardiomyopathy (HTCM) in a more realistic setting.

## Methods

This study was approved by institutional review board of our hospital and was conducted in compliance with institutional human research policy. All of the participants in the study gave written informed consent before enrollment. From August 2018 to January 2021, 185 HTCM patients and 112 healthy normal controls (N) were enrolled in this study, as shown in Table 1.

**Table 1:** Import data

Imported Data	
Original Data: Total = 297 subjects, 1793 videos	
Healthy (N)	Hypertensive (HTCM)
Total = 112 (707 videos)	Total = 185 (1086 videos)
Training dataset	Test dataset
Total = 247 (1489 videos)	Total = 50 (304 videos)

A cohort of 297 subjects (1793 videos) were divided into 247 subjects as the training dataset, and 50 subjects as the test set, in an approximately 5:1 ratio (*N* = normal, *HTCM* = hypertensive cardiomyopathy). 93 healthy controls and 154 hypertensive subjects were assigned to the training dataset, and the remaining were allocated to the test dataset. Datasets were strictly divided by subjects rather than by videos

### Inclusion and exclusion criteria

The inclusion criteria were described as follows: the diagnosis met the criteria of Chinese guidelines for the prevention and treatment of HTN (revised version in 2018), systolic blood pressure ⩾ 140 mmHg and/or diastolic blood pressure ⩾ 90 mmHg (Note: 1 mmHg = 0.133 kPa), and/or regular use of antihypertensive drugs within two weeks. The course of disease was 2–5 years, and drug treatment was insisted, LVEF ⩾ 55%, left ventricular mass index (LVMI) > 115 g/m^2^ (male), LVMI > 95 g/m^2^ (female) or relative wall thickness (RWT) > 0.42 [17]. The exclusion criteria were summarized as follows: arrhythmia, congenital heart disease, coronary heart disease, valvular disease, cardiomyopathy, and secondary hypothyroidism. All subjects included in both groups were characterized by the sinus rhythm.

### Data curation

The echocardiographic data set used in this study was collected at Henan Provincial People’s Hospital (Zhengzhou, China) by the GE Ultrasound Vivid E95 in Digital Imaging and Communications in Medicine (DICOM) format. We deployed the pydicom https://pydicom.github.io/, a pure Python package, to access video frames in the source DICOM data. The data set was a decoded pydicom object in off-the-shelf functions or software to convert private tag data in DICOM files into numerical arrays that were unavailable. Nonsensible echocardiographic videos were excluded by a trained image-based binary CNN as input the clear and informative frames of apical four chamber view and other meaningless or rather noisy, blurred frames. Simultaneously, we observed long-time videos, specifically those lasting for more than 1000 frames, and were susceptible to contain undesirable meaningless frames when clinicians aimed to seek for the right view of one particular cardiac view or to put the transducer aside and do something else. Along with the tremendous calculation requirement for 3-D convolutions,videos with no more than 300 frames were kept.

### Deep learning model

**Fig. 1:**
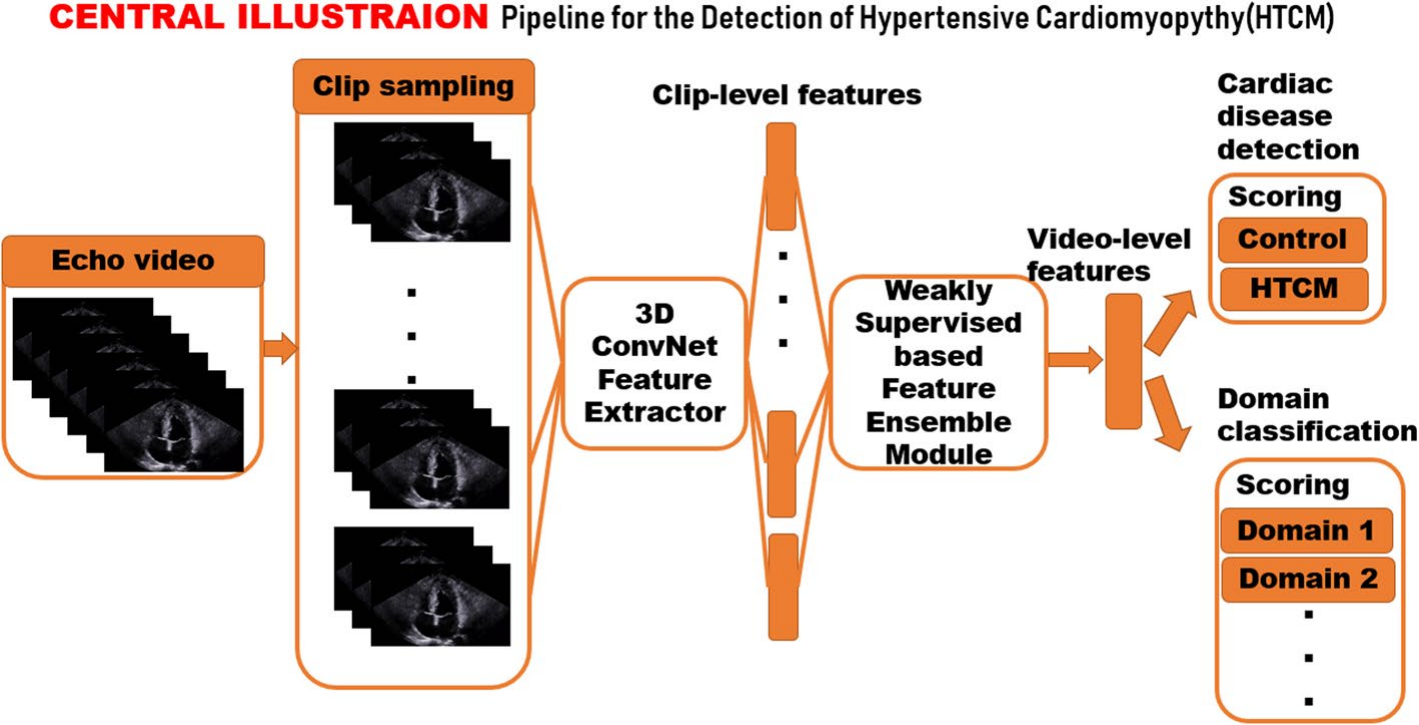
Overall pipeline for detection of hypertensive cardiomyopathy. Two sampling methods are compared (uniform sampling and cardiac cycle-based sampling), and the latter is favored. The cardiac cycle-based sampling further requires a trained ES/ED timing detection neural network based on bi-directional LSTM and 3D spatiotemporal convolutions, which is illustrated in Fig. 2

**Fig. 2:**
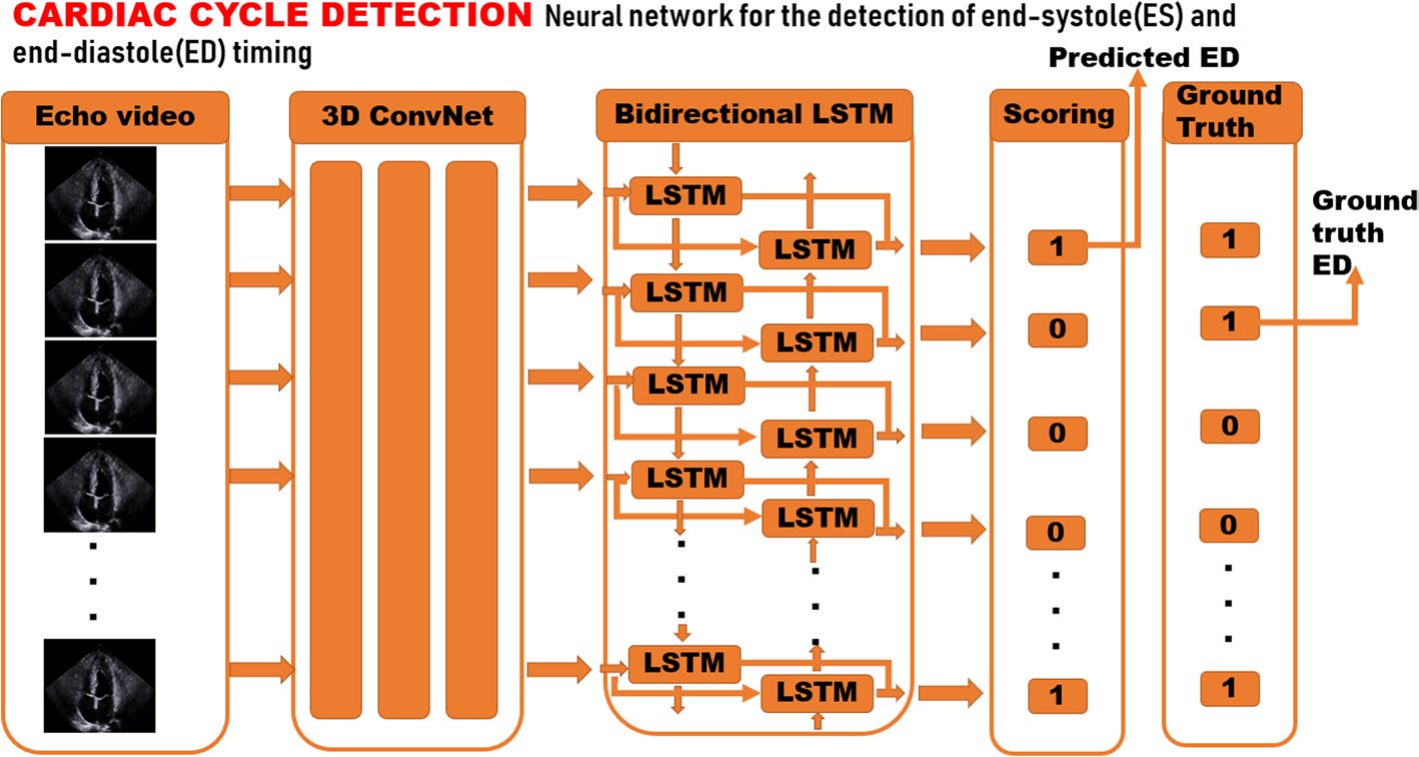
Cardiac cycle-based detection model. ES and ED timing can be determined by the last frame in consecutive systole and diastole durations, respectively. Consequently, the last’0’ in a consecutive’0’ s and the last’1’ in a consecutive’1’ s indicate end-systole and end-diastole timing, respectively

Diagnosis of an echocardiographic video practically arises from unusual left ventricular motion of consecutive frames though an untrimmed video that mainly exhibits extremely complex motion dynamics, and the desirable infrequent motion may be trivial and subtle, accounting for small portions of the overall video. Therewithal, discriminative snippets of importance were herein supposed to be attached more attention or weight when representations of segregating proposal snippets from one echo video were assigned with different probabilities and constituted a final weighted representation for downstream cardiac disease detection and domain classification, as shown in Fig. 1. The beat-to-beat based sampling could detect every cardiac cycle in the video through a trained ES/ED timing detection model (Fig. 2), and a clip comprising of 32 frames starting from end-diastole timing was selected, which was found sufficient to cover a complete cardiac contraction and dilatation process when frame per second (fps) of the GE Ultrasound Vivid E95 system (GE Healthcare, Chicago, IL, USA) was set to 30 and the frame rate of clips was 2.

### MIL

**Fig. 3:**
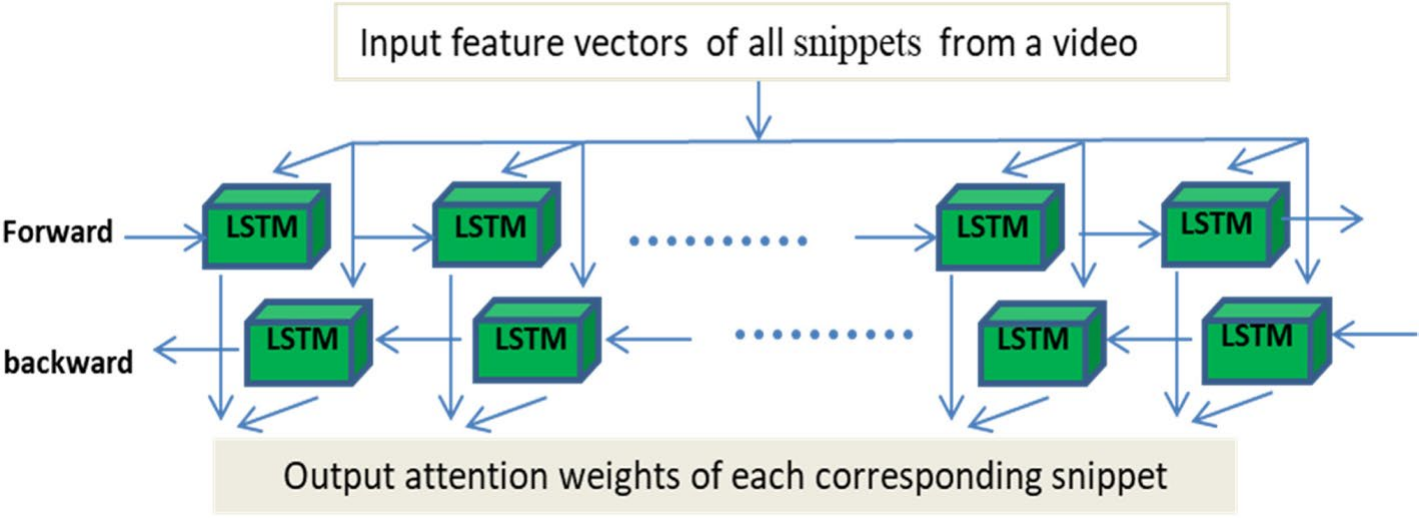
The multiple instance learning (MIL) based feature ensemble module in our proposed pipeline

The MIL can perfectly fit medical problems, such as pathological diagnosis using whole-slide imaging (WSI), due to the huge size and the lack of pixel-level annotations. The MIL attention-based method assigns the contribution of each sliced patch by introducing trainable parameters [18–22]. Apart from the independent and identical distribution hypothesis made by the classical MIL, the representations from proposal snippets are temporally correlated, and thus, the bidirectional LSTM is simply introduced to model temporal-dependent attention. The representation ensemble module (Fig. 1) sequentially outputs an ordered list of weights for clip representations and converts clip-level representations to a video-level representation. The multiple instance learning (MIL) based feature ensemble module in our proposed pipeline is shown in Fig. 3.

### Echocardiography examination

The LVEF was calculated by Simpson’s biplane method at apical views. The left ventricular end-diastolic diameter (LVDd), left ventricular end diastolic volume(LVEDV), left ventricular end-systolic volume(LVESV), interventricular septum thickness (IVST), left ventricle posterior wall thickness (LVPWT), and left ventricle mass (LVM) were measured by conventional M-mode echocardiography in all patients. The longitudinal peak strain dispersion (PSD) and global longitudinal strain (GLS) were acquired using commercially available analysis workstation (EchoPAC; GE Healthcare). All the measurements were conducted by two experienced sonographers, and all parameters were measured for three times to take the average value.

### Domain adversarial neural networks

Acquisition of echocardiographic videos of desirable standard cardiac views consists of a multitude of parameters, even varying within the same lab over time mainly because of encounters’ attributes and sonographers’ immediate preferences, resulting in a significant variation in image quality that hinders the generalization of deep learning algorithms. It is not uncommon that practitioners’ preferences are used to adjust the transducer and to acquire a high-quality echo change over time, which may further affect the clinical diagnosis. In the majority of cases, implicit approaches, such as data augmentation, are employed intensely, extending accessible training data domain to a considerable degree. Nevertheless, medical artifacts can unnecessarily lose subtle, while discriminative information in a clinical manner. As far as the echocardiogram is concerned, introduction of noises or pixel jittering may reduce the informative effect of slim mitral valve in the apical four chamber views. Domain adversarial neural networks [23] remove the domain information from the model representation by assuming that all frames extracted from an echocardiographic video originate from the same unique data distribution, and thus, constitute a domain and bifurcating from the baseline network (Table 1), in order to train a domain classification model that is less discriminative to the domains.

### Experimental details

**Fig. 4:**
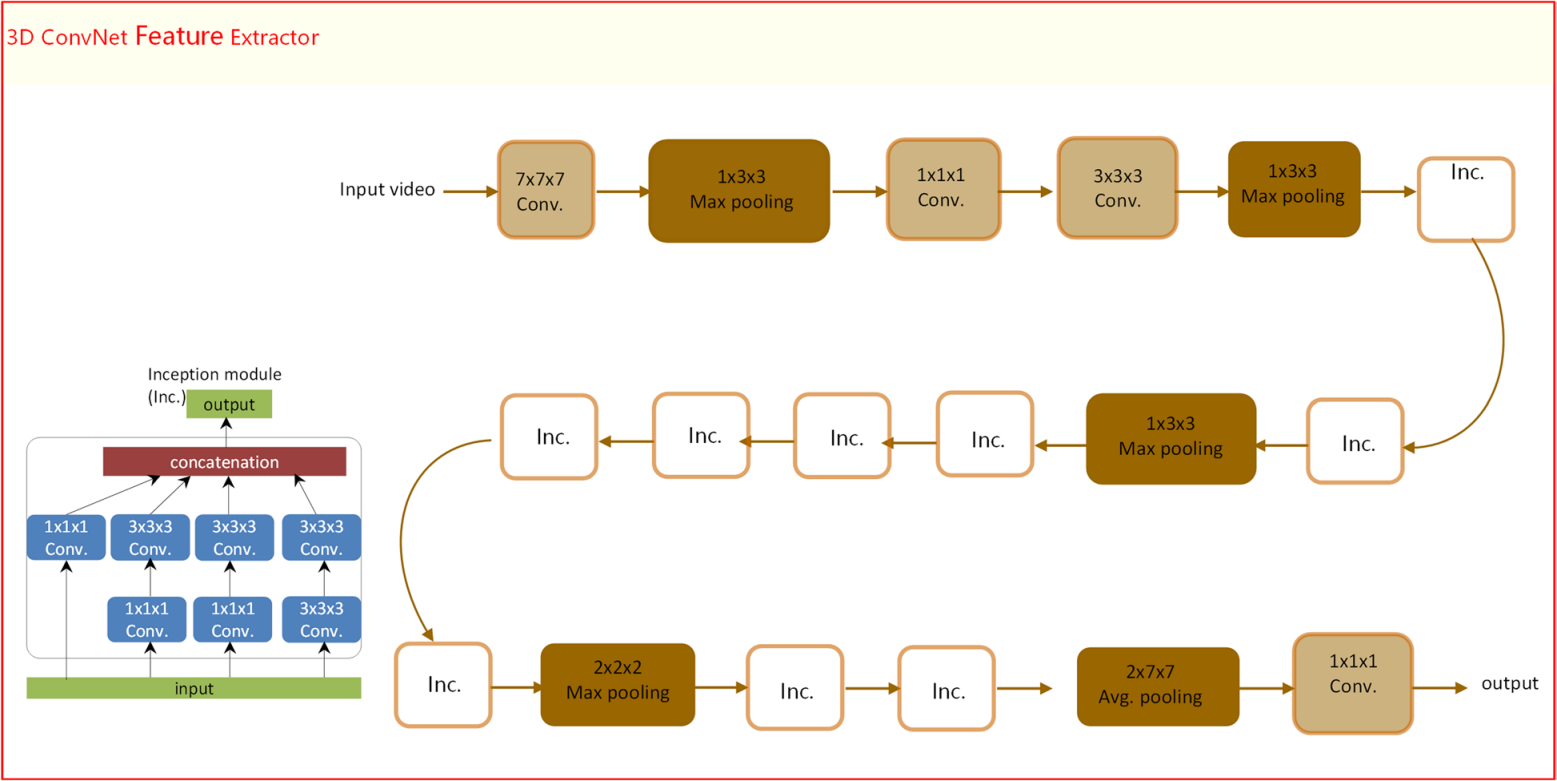
The architecture of the 3D ConvNet feature extractor used in our proposed pipeline

The pre-trained non-local I3D model [24, 25] on Kinetics-400 data set [26] served as the 3D ConvNet feature extractor (Fig. 1). The architecture of the 3D ConvNet feature extractor is illustrated in Fig. 4. During training, we decomposed one echocardiographic video into proposal snippets by the trained ES/ED timing detection model, forwarded these snippets through latter 3-D ConvNet feature extractor, and weakly supervised feature ensemble module to make the final prediction in cardiology and domain classification in an appealing end-to-end manner. The input image size was 224 × 224 by convention and the sampling rate was 2 by default. We treated every video as an individual domain, and thus, the number of domain classes was equal to the number of all videos in training and testing datasets. The training strategy was that for each iteration, a training video was firstly propagated through the whole pipeline to deliver the predicted cardiac disease class and the predicted domain class, and another video was randomly selected from the whole data set that was re-forwarded to produce only the predicted domain class, in order to include domains in test data set. The aforementioned three predicted outputs were compared with corresponding ground truth labels to yield the loss, and the PYTORCH [27] framework could back propagate and optimize the parameters once. Uniform training was employed to alleviate the data imbalance effect by training the same number of healthy and hypertensive videos for one epoch. The parameters for the HTCM detection model shown in Fig. 1 is listed as follows: the stochastic gradient descent optimizer with an initial learning rate of 0.0001, cosine decay learning rate strategy [28], weight decay of 4e-5, dropout of 0.5 [29], label smoothing of 0.1 [30], and batch size of 1 was trained for 100 epochs, accounting for a total of 50000 iterations using a Tesla V100 GPU (NVIDIA Corp., Santa Clara, CA, USA). During inference, in order to completely evaluate the performance metrics, the experiments were carried out on both the subject classification task (subject based) and the video classification task (video based). We first run the algorithms on all the individual videos to get the video classification results, and then average the classification results (the outputs of the last layer in the deep neural networks) with a same subject to get the subject classification results. Besides, only apical 4-chamber view videos were used.

### Statistical analysis

Python scikit-learn [31] library was utilized for statistical analysis. Continuous variables are expressed as mean ± standard deviation when following the normal distribution, or the median (interquartile range) for abnormal distribution. Categorical variables were represented by numbers and percentage. Comparison between hypertensive cardiomyopathy group and normal group was carried out by the two-sample t-test if normality and homogeneity of variance were both satisfied, t’-test when only homogeneity of variance was met, or the Wilcoxon signed-rank test in case of satisfying normal distribution. Receiver operating characteristic (ROC) curves are used to assess the accuracy of a diagnostic test. We use the trapezoidal rule to calculate the areas under the ROC curves (AUCs) directly. The accuracy (ACC), sensitivity (SEN), specificity (SPE), positive predictive value (PPV), negative predictive value (NPV), positive likelihood ratio(PLR), and negative likelihood ratio(NLR) were calculated. A* p*-value < 0.05 was considered statistically significant.

## Results

A data set of HTCM (185 patients, 1086 videos) and N (112healthy subjects, 707 videos) is characterized in Table 1 In addition, 31 HTCM patients and 19healthy subjects were randomly selected and assigned to the test data set, in order to improve the generalization ability of possible methods or models, and the remaining subjects were assigned to the training data set. Videos from the same subject were placed in the same split. Patients' clinical data are summarized in Table 2. There were no significant differences in age, gender, height, and surface area between the HTCM group and the N group (*P* > 0*.*05).

**Table 2:** Comparison of clinical and ultrasonic parameters

	HTCM	N	Statistics	*P* value
Age (years old)	44.91 ± 12.74	41.29 ± 13.70	*T* = -2.053	*P* = 0.041
Male	55(55%)	42(42%)	χ^2^ = 3.529	*P* = 0.060
Height (cm)	166.28 ± 89.06	165.97 ± 8.39	*T* = -0.268	*P* = 0.789
Weight (kg)	68.63 ± 11.67	65.26 ± 10.20	*T* = -2.308	*P* = 0.022
Surface area(m^2^)	1.86 ± 0.185	1.81 ± 0.169	*T* = -1.852	*P* = 0.065
BMI(kg/m^2^)	24.76 ± 3.28	23.59 ± 2.39	*T* = -3.054	*P* = 0.003
Systolic pressure (mmHg)	148.48 ± 20.92	117.80 ± 11.40	T = -13.547	*P* = 0.000
Diastolic pressure (mmHg)	93.03 ± 13.03	75.74 ± 5.58	T = -12.794	*P* = 0.000
LVEDV(ml)	108.18 ± 29.32	79.76 ± 17.35	T = -8.875	*P* = 0.000
LVESV(ml)	40.48 ± 14.50	28.20 ± 6.83	T = -8.052	*P* = 0.000
IVST(cm)	1.20 ± 0.11	0.96 ± 0.08	T = -18.609	*P* = 0.000
LVPWT(cm)	1.14 ± 0.10	0.93 ± 0.10	T = -16.734	*P* = 0.000
LVDd(cm)	4.67 ± 0.46	4.58 ± 0.32	T = -1.714	*P* = 0.088
LVM(g)	206.45 ± 47.19	148.37 ± 33.77	T = -10.572	*P* = 0.000
LVMI(g/m2)	110.78 ± 20.28	81.20 ± 13.33	T = -12.859	*P* = 0.000
RWT(cm)	0.51 ± 0.06	0.41 ± 0.02	T = -17.056	*P* = 0.000
LVEF(%)	63.38 ± 5.18	64.60 ± 4.14	T = 1.956	*P* = 0.052
GLS(%)	-17(-20,-16)	-21(-22.5,-20)	Z = -7.611	*P* = 0.000
PSD	49(41.5,60)	34(26,39)	*Z* = -7.399	*P* = 0.000

*HTCM* Hypertensive cardiomyopathy, *N* normal, *BMI* Body mass index, *LVEDV* Left ventricular end diastolic volume, *LVESV* Left ventricular end systolic volume, *IVST* Interventricular septum thickness, *LVPWT* Left ventricle posterior wall thickness, *LVDd* Left ventricular end-diastolic diameter, *LVM* Left ventricle mass, *LVMI* Left ventricular mass index, *RWT* Relative wall thickness, *LVEF* Left ventricular ejection fraction, *GLS* Global longitudinal strain, *PSD* Longitudinal peak strain dispersion

The values of body mass index (BMI) and weight significantly increased in the HTCM group compared with those in the N group (*P* < 0*.*05). The degree of the blood pressure in the HTCM group was significantly higher than that in the N group. The echocardiographic data are presented in Table 2. Besides, IVST and LVPWT were thinner in the N group than those in the HTCM group (*P* < 0*.*05). There were no significant difference in the LV size and EF between the two groups (*P* > 0*.*05). The values of LVM, LVMI, and RWT were significantly reduced in the N group compared with those in the HTCM group (*P* < 0*.*05). A significant difference was found between the HTCM group and the N group in GLS and PSD (*P* < 0*.*05).

The classification results in the test data set (subject-based) are summarized in Table 3. Almost all metrics presented in our proposed algorithm were more significant than those of the vanilla I3D approach. Specifically, our method achieved accuracy of 92%, AUC of 0.90, sensitivity of 97%, and specificity of 84%, whereas the vanilla I3D approach yielded accuracy of 90%, AUC of 0.89, sensitivity of 94%, and specificity of 84%(Table 3). In the classification results for test data set (video-based), compared with the other methods, the AUC of our proposed algorithm reached 0.90 for predicting the presence of hypertensive cardiomyopathy, with 0.97 sensitivity, 0.84 specificity, 0.91 PPV and 0.94 NPV (Table 4).

**Table 3:** Classification results for test dataset (subject-based)

	ACC	AUC	95%CI	SEN	SPE	PPV	NPV	PLR	NLR
Image-based method (random)	0.83	0.82	0.77–0.87	0.89	0.75	0.81	0.85	3.62	0.14
Image-based method (ES/ED)	0.79	0.77	0.72–0.83	0.89	0.66	0.76	0.83	2.60	0.17
Vanilla I3D	0.85	0.85	0.80–0.89	0.90	0.80	0.84	0.87	4.42	0.13
Ours	0.86	0.85	0.81–0.90	0.92	0.79	0.84	0.89	4.45	0.11

*ACC* Accuracy, *AUC* Area under receiver operating characteristic (ROC) curve, *SEN* Sensitivity, *SPE* Specificity, *PPV* Positive predictive value, *NPV* Negative predictive value, *PLR* Positive likelihood ratio, *NLR* Negative likelihood ratio, *CI* Confidence interval

**Table 4:** Classification results for test dataset (video-based) set

	ACC	AUC	95%CI	SEN	SPE	PPV	NPV	PLR	NLR
Image-based method (random)	0.86	0.83	0.70–0.95	0.97	0.68	0.83	0.93	3.06	0.05
Image-based method (ES/ED)	0.88	0.85	0.73–0.97	0.97	0.74	0.86	0.93	3.68	0.04
Vanilla I3D	0.90	0.89	0.78–0.99	0.94	0.84	0.91	0.89	5.92	0.08
Ours	0.92	0.90	0.81–1.00	0.97	0.84	0.91	0.94	6.13	0.04

*ACC* Accuracy, *AUC* Area under receiver operating characteristic (ROC) curve, *SEN* Sensitivity, *SPE* Specificity, *PPV* Positive predictive value, *NPV* Negative predictive value, *PLR* Positive likelihood ratio, *NLR* Negative likelihood ratio, *CI* Confidence interval

In addition, the results of two image-based methods (random selection-based method [14] and ES/ED-based method [13] were also included for impartial comparison with video-based ones. The two image-based approaches, especially the ES/ED-based method [13] yielded results (video-based) comparable with the vanilla I3D approach (sensitivity: 97% (ES/ED-based method) vs. 94% (vanilla I3D), NPV: 93% (ES/ED-based method) vs. 89% (vanilla I3D), Table 4).

Besides, the huge gap in classification results in the test data set (video-based) between the image-based methods and the video-based methods, as summarized in Table 4, demonstrates a greater spatiotemporal feature extraction ability of the 3-D ConvNet feature extractor (AUC: 0.83 for image-based method (random), 0.85 for ES/ED-based method, 0.89 for vanilla I3D, and 0.90 for our pipeline; Table 4).

**Fig. 5:**
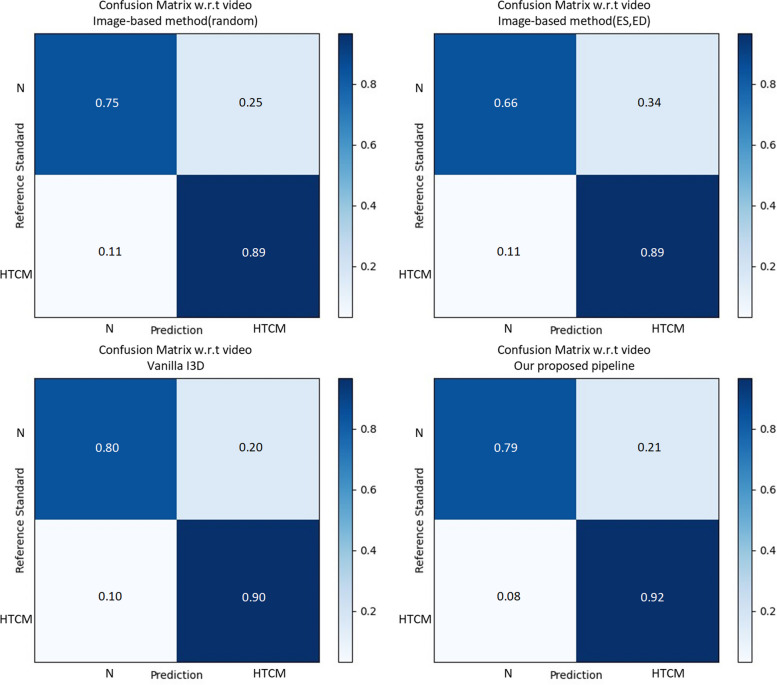
Confusion matrix for classification results in the test dataset (subject-based)

**Fig. 6:**
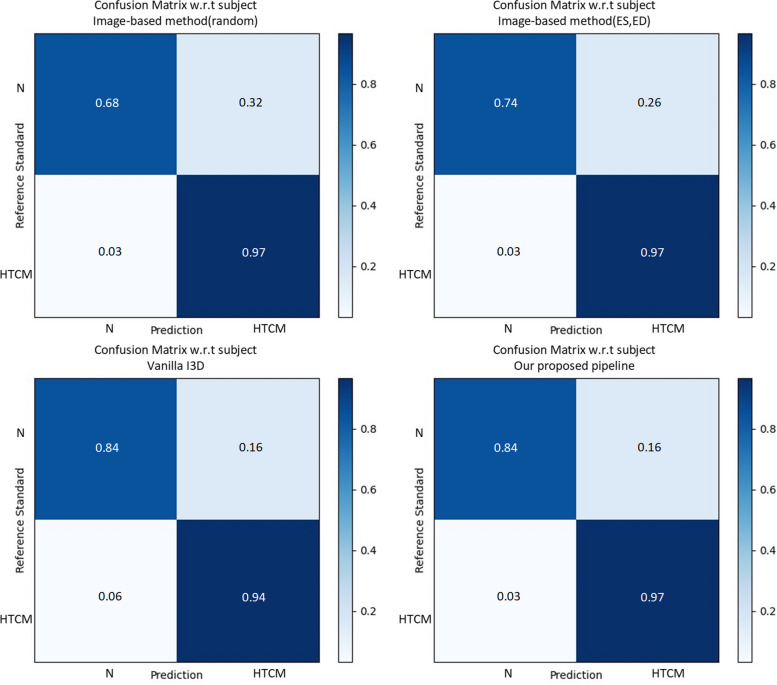
Confusion matrix for classification results in the test dataset (video-based)

**Fig. 7:**
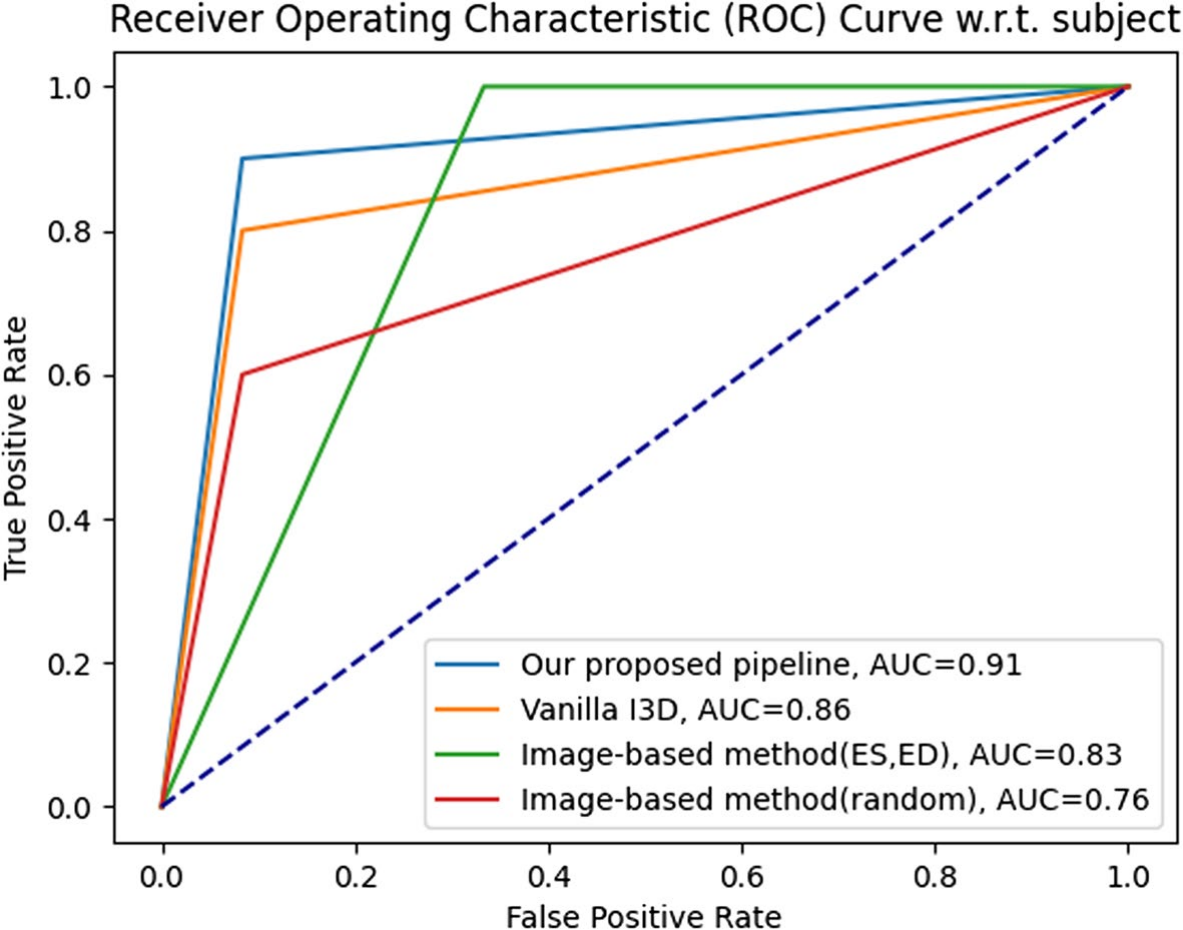
ROC curve for classification results in the test dataset (subject-based)

**Fig. 8:**
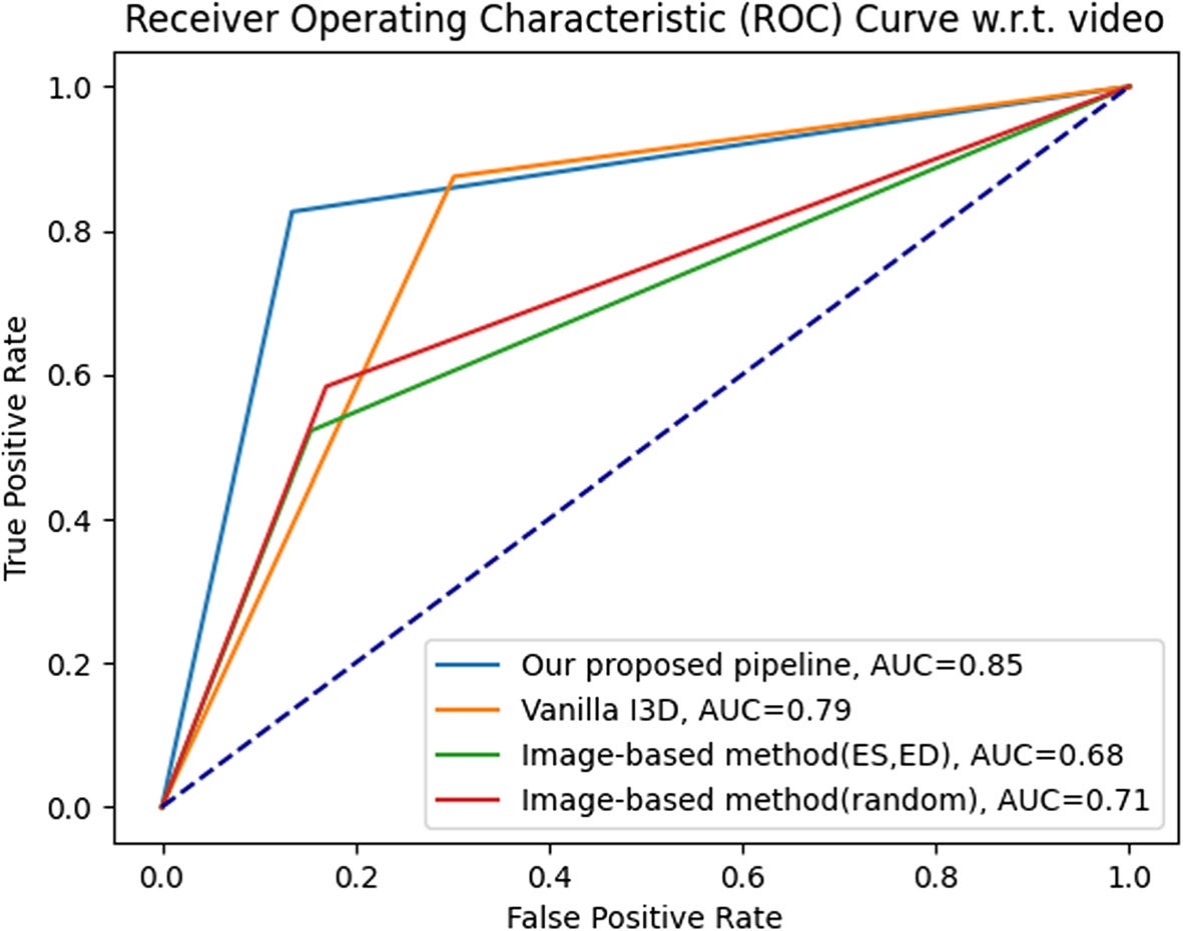
ROC curve for classification results in the test dataset (video-based)

In general, confusion matrix is a very popular measure used, while solving classification problems. Confusion matrices formulated in our method are presented in Figs. 5 and 6. In addition, the results of the ROC curve analysis were used to assess the ability of the above-mentioned methods to detect the hypertensive cardiomyopathy (Figs. 7 and 8). From these results, our proposed algorithm can provide higher AUC than all the other baselines (*p* < 0.001).

## Discussion

HTN has been regarded as an independent risk factor for cardiovascular disease, and the left ventricular remodeling and systolic dysfunction were mainly targeted. HTN patients could maintain normal ejection function in the preclinical period, while the subclinical morphological changes may occur before functional alterations [32]. As shown in Table 2, the values of LVM, LVEDV, LVESV, IVST, LVPWT, LVMI, and RWT increased in the HTCM group compared with those in the N group, indicating that the impaired cardiovascular structure was already present in the early stages of HTN. The GLS value in the HTCM group decreased compared with that in the N group, while the PSD value in the HTCM group increased. The myocardial strain analysis could be applied to evaluate cardiac dysfunction with preserved ejection fraction.

Human assessment of cardiac function clinically depends on laborious and time-consuming calculation of LVEF, PSD, and global longitudinal strain from a limited sampling of cardiac cycles, and it is accompanied by a noticeable inter- or intra-observer variability regardless of years of training [33, 34]. In the present study, the time–space model was established for dynamic videos, and it could better reflect the true cardiac motions. This method was unsupervised with no intra-observer error. While our multi-instance pipeline is a problem-specific extension of the vanilla I3D approach [25], and the vanilla I3D is intrinsically an instance-level fully supervised approach designated for trimmed videos. Despite instance-level ground truth for snippets in one video that was unavailable in our scenario and only bag-level label was known for all videos, the vanilla I3D could yield satisfactory results because of huge number of trainable parameters constituting a 3-D CNN and early stopping technique that was constantly adaptive to the test data set. Results of this diagnostic study demonstrated the capability of weakly supervised multi-instance video-based deep learning architecture to detect hypertensive cardiomyopathy in echocardiographic videos with variable durations. This finding is important in our proposed pipeline that training was performed by only reference labels per video, whereas implicit approaches require frame-level segmentation annotation for each left ventricle and video-level LVEF [14]. Because of the time and resources required for annotating echocardiographic videos, fewer requirements for these annotations would facilitate augmentation of data set, particularly for the future research on video analysis because deep learning algorithms are quite beneficial when dealing with learning from large amounts of unsupervised data.

As shown in Tables 3 and 4, the attention mechanism of our proposed workflow directs the information flow and forces the network to identify discriminative snippets. The attention mechanism is more likely to assign larger weights to snippets of lower LVEDVs or LVESVs for a video from hypertensive cardiomyopathy category, whereas more attention is in favor of snippets of higher LVEDVs or LVESVs for a video from a healthy class. It mimics the actual diagnostic process performed by sonographers and cardiologists via examining the most discriminative snippet to some extent, and the attention mechanism allows for tolerance of insignificant cardiac cycles by assigning lower weights.

However, our proposed pipeline may not surpass the remainder in all evaluation metrics (specificity: 0.80 (vanilla I3D) vs. 0.79 (ours), Table3), which could be related to the early stopping strategy, in order to acquire better subject-based metrics rather than video-based metrics. The clinical parameters presented in Tables 3 and 4 might not be very precise when the process of compressing echo videos into DICOM format loses information and thus blurs the margins of left ventricle. The variable depth zooms in the echocardiogram can achieve a more operator favorable standard view, resulting in clinically unreasonable difference in the left ventricular volumes (LVEDV and LVESV) from a video to another video.

In the present study, it was found that the deep learning model could be used to automatically identify the dynamic video of echocardiography, which could better reflect the cardiac motion. This method might not be the same as the one taken by a human reader, and it could be utilized for detection of hypertensive cardiomyopathy, which could identify some views in echocardiographic video that are untypically used by clinicians, while being informative for an automated system. The automatic and rapid recognition results achieved by artificial intelligence could assist sonographers in abnormal echocardiographic videos, indicating the necessity of further detailed measurement and analysis. The results could provide a basis for a data-driven platform that could identify patients most likely responding to specific attention.

## Limitations

Although this study possesses some advantages for diagnosing hypertensive cardiomyopathy, it still has some limitations. First, there was an unsolved echocardiogram information loss in the data compression and extraction processes. The trained model may only be applicable to data distribution in such setting and it may fail with respect to the clearer echocardiogram, while domain adaptation is worthy of consideration. Second, our proposed pipeline is an explicit method, taking an echo video as input and a classification model as output, without exporting clinically interpretable parameters, such as LVEDV, LVEDS, etc. However, the specificity of attention-based method exhibited interpretability in a different manner (Tables 3 and 4), and it can implicitly discover the most representative snippet, facilitating estimation of potential downstream clinical parameters. Third, the overall data set was relatively small compared with conventional data set in deep learning [15], and we did not assess the cross healthcare system or reliability for the ultrasound system using the proposed model in the lack of associated data set. Empirically, diagnosis of cardiac disease depends on a variety of factors, such as enquiries, medical history, echocardiogram, blood pressure, pertinent biochemical indices, etc. In this study, the deep learning algorithm detected the HTCM merely from echo videos, and additional studies can involve diagnostic results that practitioners derived from only echo data to conduct a righteous comparison with our fully automated method in terms of precision of the CPU time. Multi-modal learning is worthy of further investigation due to the existence of various sources influencing the final diagnosis.

## Conclusions

In summary, in order to the video-based detection of the hypertensive cardiomyopathy with the normal ejection fraction, a new CNN was herein developed that effectively incorporated temporal correlated multi-instance learning, domain adversarial neural networks, and end-systole and end-diastole attributes of echo video to enhance the discriminative effect of the most representative snippet. The presented MIL-based model showed to be applicable to detection of cardiac disease. Furthermore, the findings may promote clinical interventions for cardiac disease and reduce sonographers' workload.

## Data Availability

All data and methods are available from the corresponding author by request.
